# Complex Carbohydrates and Glycoconjugates: Structure, Functions and Applications

**DOI:** 10.3390/ijms222212219

**Published:** 2021-11-12

**Authors:** Alexander O. Chizhov

**Affiliations:** N.D. Zelinsky Institute of Organic Chemistry, Russian Academy of Sciences, 119991 Moscow, Russia; chizhov@ioc.ac.ru

The study of carbohydrates has a long history: for two centuries, the researches performed the way from “sweet matter” to glycomics. The complexity of cabohydrates analyzed in such studies grew enormously: the term “complex carbohydrates” includes large oligosaccharides (dozens of carbohydrate units) and polysaccharides, both regular or irregular. Carbohydrate studies were inevitably expanded to adjacent areas which is reflected by the general term “glycoconjugate” (a compound, in which carbohydrate molecule part(s) is/are covalently bonded with non-carbohydrate part(s)).

Nowadays, carbohydrate science is an integral part of molecular biology, along with genomics and proteomics. It includes structural studies of glycans, glycoproteins, proteoglycans, glycolipids, low-molecular and complex glycosides of plant, animal, fungal, and bacterial origin. Supramolecular structural studies such as cell wall reconstruction are developed. The functional studues of carbohydrates concern molecular recognition such as carbohydrate–lectin or glycoside–enzyme interactions, cell recognition (normal and in pathologies), viral adhersion and penetration, oligo- and polysaccharide biosynthesis, and many other phenomena. In such studies, artificial carbohydrate-containing molecular probes (synthetic glycoconjugates) are widely used now.

The structural and functional studies are extremely stimulated by the progress of instrumental methods, especially in chromatography, electrophoresis, multidimensional NMR spectrometry, high-resolution mass spectrometry, and surface plasmon resonance. Hyphenated techniques such as HPLC-MS are an integral part of modern carbohydrate analysis methodology. Modeling of complex carbohydrates structures using molecular mechanics and quantum chemical calculations is widely used to expand our understanding of their functioning. Big Data analysis became inevitable due to enormous the volume of information relating to glycochemistry and glycobiology (glycoinformatics).

The diversity of modern glyco studies both in terms of subjects and methods is clearly illustrated by the variety of papers published in this Special Issue and summarized below.

Rational design of a glycoconjugate vaccine against group A *Streptococcus* (GAS), major cause of pharyngitis and impetigo, has been proposed by Di Benedetto et al. Group A Carbohydrate (GAC) conjugated to an appropriate carrier protein (GAS Streptolysin O (SLO), SpyCEP and SpyAD) was investigated as an attractive vaccine candidate. Conjugation to the polysaccharide had a negative impact on the anti-protein responses, especially in terms of functionality as evaluated by an IL-8 cleavage assay for SpyCEP and a hemolysis assay for SLO. After selecting CRM_197_ as a carrier, optimal conditions for its conjugation to GAC were identified through a Design of Experiment approach, improving process robustness and yield [1]. 

Glucanosyltransglycosylases are the key enzymes in construction of yeast cell wall (CW). Glucan linked to proteins is a natural mega-glycoconjugate (mGC) playing the central role as a structural component of a yeast CW. The main ncGTGs Bgl2 and Scw4 have phosphorylated and glutathionylated residues and are represented in CW as different pools of molecules having various firmness of attachment. Identified pools contain Bgl2 molecules with unmodified peptides but differ from each other in the presence and combination of modified ones, the presence or absence of other CW proteins. Glutathione affects Bgl2 conformation, probably resulting in the mode of its attachment and enzymatic activity. Bgl2 from the pool of unmodified and monophosphorylated molecules demonstrates the ability to fibrillate after isolation from CW. Functioning of ncGTGs in CW can be controlled by reversible post-translational modifications and facilitated due to their compact localization [2].

Collision-induced decay (CID) of protonated, ammoniated, and metallated molecules of cyclic *N*-substituted oligo-β-(1→6)-D-glucosamines differing in cycle size and *N*-acyl substituents results in a cleavage of glycosidic bonds of a cycle. In some cases, fragmentation of amide side chains is possible. If labile fragments in substituents (e.g., carbohydrate chains) are present, a decay of the cycle and an elimination of labile fragments are of comparable possibility. It was found that in some cases rearrangements with loss of an internal carbohydrate residue (IRL), or an internal part of a side chain, are feasible [3].

Endocytosis and trafficking of heparan sulfate proteoglycans (HSPG) in triple-negative breast cancer cells were studied by Mandarini et al. [4]. HSPG endocytosis could affect two important phenomena of cancer development: cell migration and nourishment. Using NT4 as an experimental tool mimicking heparin-binding ligands, they studied endocytosis and trafficking of HSPGs in a triple-negative human breast cancer cell line, MDA-MB-231. The peptide entered cells employing caveolin- or clathrin-dependent endocytosis and macropinocytosis, in line with what is already known about HSPGs. NT4 then localized in early and late endosomes in a time-dependent manner. The peptide had a negative effect on CDC42-activation triggered by EGF. The effect was explained considering NT4 a competitive inhibitor of EGF on HS that impairs the co-receptor activity of the proteoglycan, reducing EGFR activation. Reduction in the invasive migratory phenotype of MDA-MB-231 induced by NT4 was ascribed to this effect. RhoA activation was damped by EGF in MDA-MB-231. Indeed, EGF reduced RhoA-GTP and NT4 did not interfere with this receptor-mediated signaling. The peptide alone determined a small but solid reduction in active RhoA in breast cancer cells. This result supports the observation of a few other studies, showing direct activation of the GTPase through HSPG, not mediated by EGF/EGFR.

Molecular dynamics models of nonsulfated glycosaminoglycans (GAGs) were constructed by Whitmore et al. [5]. An algorithm which applies conformational parameters (i.e., all bond lengths, bond angles, and dihedral angles) from molecular dynamics (MD) simulations of nonsulfated chondroitin GAG 20-mers tested successfully to construct 3-D atomic-resolution models of nonsulfated chondroitin GAGs of arbitrary length was applied to hyaluronan and nonsulfated forms of dermatan, keratan, and heparan and expanded the authors’ database of MD-generated GAG conformations. They showed that individual glycosidic linkages and monosaccharide rings in 10- and 20-mers of hyaluronan and nonsulfated dermatan, keratan, and heparan behave randomly and independently in MD simulation. The algorithm efficiently constructed conformational ensembles of GAG 200-mers. 

In order to reveal applicability of calculations for automated carbohydrate database filling, six empirical force fields were tested. They were probed on eleven disaccharide molecules containing representative structural features from widespread classes of carbohydrates. The accuracy of each method was queried by predictions of nuclear Overhauser effects (NOEs) from conformational ensembles obtained from 50 to 100 ns molecular dynamics (MD) trajectories and their comparison to the published experimental data. Using various ranking schemes, it was concluded that explicit solvent MM3 MD yielded non-inferior NOE accuracy with newer GLYCAM-06, and ultimately PBE0-D3/def2-TZVP (Triple-Zeta Valence Polarized) Density Functional Theory (DFT) simulations. For seven of eleven molecules, at least one empirical force field with explicit solvent outperformed DFT in NOE prediction. The aggregate of characteristics (accuracy, speed, and compatibility) made MM3 dynamics with an explicit solvent at 300 K the most favorable method for bulk generation of disaccharide conformation maps for massive database filling [6].

Human natural antibodies recognizing glycan Galβ1-3GlcNAc (Le^C^) were isolated from donors’ blood using Le^C^-Sepharose. It is known that the level of human natural antibodies of immunoglobulin M isotype against Le^C^ in patients with breast cancer is lower than in healthy women. The isolated antibodies recognize the disaccharide but do not bind to glycans terminated with Le^C^, which implies the impossibility of binding to regular glycoproteins of non-malignant cells. The avidity (as dissociation constant value) of antibodies probed with a multivalent disaccharide is 10^−9^ M; the nanomolar level indicates that the concentration is sufficient for physiological binding to the cognate antigen. Testing of several breast cancer cell lines showed the strongest binding to ZR 75-1. Only 7% of the cells were positive in a monolayer with a low density, increasing up to 96% at highest density. The enhanced interaction (instead of the expected inhibition) of antibodies with ZR 75-1 cells in the presence of Galβ1-3GlcNAcβ disaccharide indicates that the target epitope of anti-Le^C^ antibodies is a molecular pattern with a carbohydrate constituent rather than a glycan [7].

Rearrangement of the cellulose-enriched cell wall in flax phloem fibers over the course of the gravitrope reaction was studied by Ibragimova et al. [8]. Profound changes in cell wall organization are detected by microscopy in the phloem fibers of flax (*Linum usitatissimum*) during the restoration of the vertical position of the inclined stems. Biochemical analysis revealed a slight increase in the content of pectins in the fiber cell walls of gravistimulated plants as well as an increase in accessibility for labeling non-cellulosic polysaccharides. The presence of galactosylated xyloglucan in the gelatinous cell wall layer of flax fibers was demonstrated, and its labeling was more pronounced in the gravistimulated plants. An increase in the crystallinity of the cellulose in gravistimulated plants along with a decrease in cellulose mobility was found with solid-state NMR. 

Artificial tetrameric lectins were constructed by Achilli et al. [9]. C-type lectin receptor (CLR)/carbohydrate recognition occurs through low affinity interactions. Nature compensates that weakness by multivalent display of the lectin carbohydrate recognition domain (CRD) at the cell surface. Mimicking these low affinity interactions in vitro is essential to better understand CLR/glycan interactions. A strategy to create a generic construct with a tetrameric presentation of the CRD for any CLR, termed TETRALEC was applied to a naturally occurring tetrameric CRD, DC-SIGNR, and compared the TETRALEC ligand binding capacity by synthetic N- and O-glycans microarray using three different DC-SIGNR constructs its natural tetrameric counterpart, the monomeric CRD and a dimeric Fc-CRD fusion. The DC-SIGNR TETRALEC construct showed a similar binding profile to that of its natural tetrameric counterpart. Differences observed in recognition of low affinity ligands underlined the importance of the CRD spatial arrangement. DC-SIGNR TETRALEC was applied to evaluate CLR/pathogens interactions. This construct was able to recognize heat-killed *Candida albicans* by flow cytometry and confocal microscopy, a so far unreported specificity of DC-SIGNR [9]. 

Aberrant glycosylation of immunoglobulin G (IgG) implicated in rheumatoid arthritis (RA) pathogenesis was investigated. Permethylated *N*-glycans of the IgG obtained in serum from 44 RA patients and 30 healthy controls were profiled using linear ion-trap electrospray ionization mass spectrometry (LTQ-ESI-MS). IgG *N*-glycosylation and rheumatoid factor levels were compared in healthy controls and RA patients. The results suggested that total IgG purified from serum of RA patients shows significantly lower galactosylation, lower sialylation and higher fucosylation levels compared with healthy controls. A positive correlation between aberrant *N*-glycosylation and rheumatoid factor level in the RA patients was observed [10].

A water-insoluble polysaccharide was isolated and purified from the biofilm produced by *Burkholderia cenocepacia* strain H111, a cystic fibrosis pathogen. The repeating unit for the water-insoluble *B*. *cenocepacia* biofilm polysaccharide was revealed as [3)-α-d-Gal*p*-(1→3)-α-d-Glc*p*-(1→3)-α-d-Gal*p*-(1→3)-α-d-Man*p*-(1→]_n_ by GLC–MS and 1D and 2D NMR spectroscopy. Molecular modelling was used, coupled with NMR Nuclear Overhauser Effect (NOE) data, to obtain information about local structural motifs which could give hints about the polysaccharide insolubility. Both modelling and NMR data pointed at restricted dynamics of local conformations which were ascribed to the presence of inter-residue hydrogen bonds and to steric restrictions. Good correlation between NOE data and calculated interatomic distances by molecular dynamics simulations validated potential energy functions used for calculations https://www.mdpi.com/1422-0067/21/5/1702 (accessed on 2 March 2020) [11].

Protective effect of α-l-hexaguluroic acid hexasodium salt (G6) against UVA-induced photoaging of human keratinocyte cells was demonstrated by Li et al. [12]. It was found that G6 localized to the mitochondria and improved mitochondrial functions. G6 increased respiratory chain complex activities, which led to increased cellular ATP content and NAD^+^/NADH ratio. G6 alleviated the oxidative stress state in UVA-irradiated cells and can regulate the SIRT1/pGC-1α pathway, which enhanced the cells’ viability and mitochondria energy metabolism. The anti-photoaging potential of G6 is directly associated with the increased level of MMP and SIRT1, which was followed by the upregulation of *pGC-1α*, *D-LOOP*, and *Mt-TFA*, and with the transcriptional activation of *NRF1/NRF2*. Thus, G6 can protect HaCaT cells from UVA-induced photo-aging via the regulation of mitochondria energy metabolism and its downstream signaling pathways.

Genome-wide analysis of whole human glycoside hydrolases was performed in silico. Analysing the evolution of these enzymes is essential for improving the understanding of glycan metabolism and function. A genome-wide analysis of whole human glycoside hydrolases was performed using the UniProt, BRENDA, CAZy and KEGG databases. Using cluster analysis, 319 human glycoside hydrolases were classified into four clusters based on their similarity to enzymes conserved in chordates or metazoans (Class 1), metazoans (Class 2), metazoans and plants (Class 3) and eukaryotes (Class 4). The eukaryote and metazoan clusters included *N*- and *O*-glycoside hydrolases, respectively. The significant abundance of disordered regions within the most conserved cluster indicated a role for disordered regions in the evolution of glycoside hydrolases. These results suggest that the biological diversity of multicellular organisms is related to the acquisition of *N*- and *O*-linked glycans [13].

Preparation of defined chitosan oligosaccharides using chitin deacetylases was discussed. Chitosan polymers with defined, non-random pattern of acetylation (PA), i.e., the sequence of acetylated and non-acetylated residues along the linear polymer are not yet available. One way in which the PA will influence the bioactivities of chitosan polymers is their enzymatic degradation by sequence-dependent chitosan hydrolases present in the target tissues. The PA of the polymer substrates in conjunction with the subsite preferences of the hydrolases determines the type of oligomeric products and the kinetics of their production and further degradation. The bioactivities of chitosan polymers will at least in part be carried by the chitosan oligomers produced from them, possibly through their interaction with pattern recognition receptors in target cells. In contrast to polymers, partially acetylated chitosan oligosaccharides (paCOS) can be fully characterized concerning their DP, *F*_A_, and PA, and chitin deacetylases (CDAs) with different and known regio-selectivities are currently emerging as efficient tools to produce fully defined paCOS in quantities sufficient to probe their bioactivities. CDA applications in forward and reverse mode used to produce all of the possible paCOS dimers, trimers, and tetramers, most of the pentamers and many of the hexamers are reviewed [14].

Approaches of chemo- and glyco-informatics to 3D structural data generation, deposition and processing with regard to carbohydrates and their derivatives are described. Databases, molecular modeling and experimental data validation services, and structure visualization facilities developed for the last five years are reviewed in [15].

Metabolism of glycosphingolipids (GSLs) and their role in the pathophysiology of lysosomal storage disorders are reviewed by Ryckman et al. [16]. Of the hundreds of unique GSL structures, anionic gangliosides are the most heavily implicated in the pathogenesis of lysosomal storage diseases (LSDs) such as Tay-Sachs and Sandhoff disease. Each LSD is characterized by the accumulation of GSLs in the lysosomes of neurons, which negatively interact with other intracellular molecules to culminate in cell death. The biosynthesis and degradation pathways of GSLs are summarized, contribution of aberrant GSL metabolism to key features of LSD pathophysiology, parallels between LSDs and neurodegenerative proteinopathies such as Alzheimer’s and Parkinson’s disease, and possible therapies for patients are discussed.

The last review is devoted to the recent advancements of microcarriers based on biopolymers, especially polysaccharides such as chitosan, chitin, cellulose, hyaluronic acid, alginate, and laminarin, for 3D cell culture and the fabrication of engineered tissues based on them. The concept of three-dimensional (3D) cell culture has been proposed to maintain cellular morphology and function as in vivo. Among different approaches for 3D cell culture, microcarrier technology provides a promising tool for cell adhesion, proliferation, and cellular interactions in 3D space mimicking the in vivo microenvironment. In particular, microcarriers based on biopolymers have been widely investigated because of their superior biocompatibility and biodegradability. Microcarriers opened avenue for fabricating engineered tissues, which is one of the cutting-edge topics in tissue engineering and regeneration medicine. The current limitations and perspectives are also discussed [17].
